# Assessment of the Role of miR‐30a‐5p on the Proliferation and Apoptosis of Hair Follicle Stem Cells

**DOI:** 10.1111/jocd.16644

**Published:** 2024-10-23

**Authors:** Yiping Wang, Wentao Wu, Risheng Wang, Jinwei Chen, Xiangping Xu, Meiqi Li, Chiyu Jia, Nian Chen

**Affiliations:** ^1^ Center of Burn & Plastic and Wound Healing Surgery, Hengyang Medical School The First Affiliated Hospital, University of South China Hengyang China; ^2^ Department of Burns and Plastic Surgery The Affiliated Zhuzhou Hospital Xiangya Medical College CSU Zhuzhou China

**Keywords:** hair loss, miR‐30a‐5p, stem cells, Wnt/β‐catenin

## Abstract

**Objective:**

To investigate the role of miR‐30a‐5p on the proliferation and apoptosis of hair follicle stem cells (HFSCs) and whether the Wnt/β‐catenin signaling pathway is involved.

**Methods:**

HFSCs derived from the vibrissa of mammary rats were obtained by enzymatic digestion, and subsequently the obtained HFSCs were treated with Lipofectamine 2000 cell transfection and divided into normal cell culture group (control), miR‐30a‐5p overexpression group (miR‐30a‐5p mimic), miR‐30a‐5p empty vector group (miR‐NC), miR‐30a‐5p inhibitor group (in‐miR‐30a‐5p), and in‐miR‐30a‐5p empty vector group (in‐miR‐NC). After transfection, the cell proliferation and apoptosis rates were examined separately. In addition, the mRNA expression of β‐catenin, proliferating cell nuclear antigen (PCNA) and apoptosis‐related genes (Bax and Bcl‐2) were examined.

**Results:**

The results of cell proliferation ability showed that in‐miR‐30a‐5p group promoted cell proliferation of HFSCs relative to other groups, along with significant upregulation of gene levels of PCNA. Apoptosis analysis indicated that apoptosis rate was reduced in the in‐miR‐30a‐5p group, and the expression of Bax was suppressed, while that of Bcl‐2 was promoted. Wnt/β‐catenin signaling pathway investigation revealed a significant increase in the levels of β‐catenin in HFSCs in the in‐miR‐30a‐5p group.

**Conclusion:**

Downregulation of miR‐30a‐5p levels inhibited HFSCs apoptosis and simultaneously promoted proliferation, furthermore, the increased expression of β‐catenin indirectly confirmed the activation of the Wnt/β‐catenin signaling pathway.

## 1 Introduction

Hair loss is one of the most common health problems, with approximately 2 billion people worldwide suffering from varying degrees of hair loss [1]. Although hair loss itself is not life‐threatening, its negative impact on the mental health of the population cannot be ignored, and psychological problems such as anxiety and depression can affect social activities and career development [2].

**FIGURE 1:**
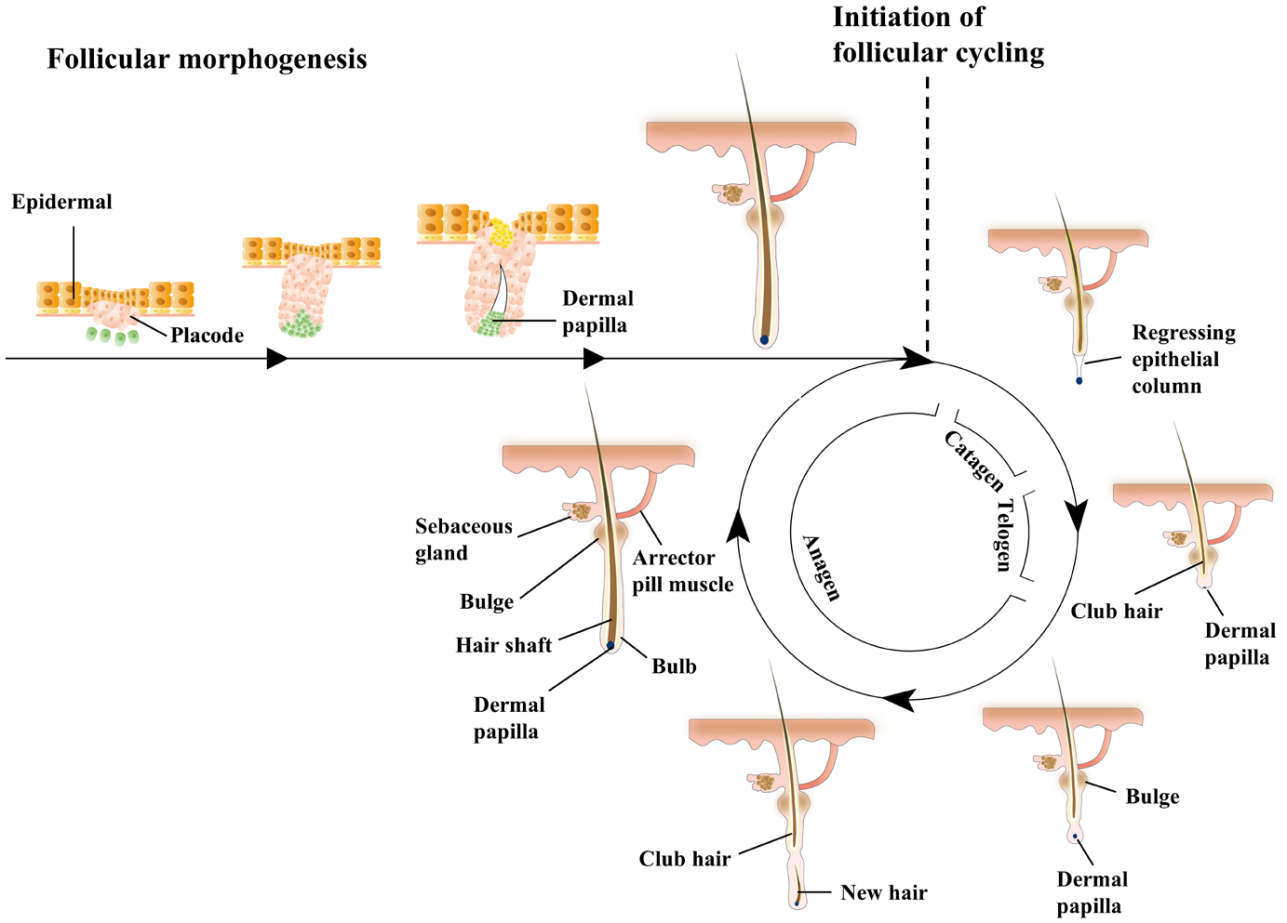
Hair follicle morphogenesis and the hair follicle cycle. Early in embryonic development, dermal cells release specific signaling molecules (e.g., Wnt signaling pathway factors) that stimulate localized thickening of epidermal cells, resulting in the formation of the placode, which grows downward to form the hair peg. The continued lengthening of the hair peg facilitates the enlargement of the bulb in the lower portion of the hair follicle. Different cell layers in the hair follicle begin to form, such as the outer root sheath (ORS), the inner root sheath (IRS), and the precursor of the hair shaft. At this stage, the bulge region, which stores HFSCs, is formed. The differentiated hair follicle enters the cyclic growth phase, that is, anagen, catagen, and telogen.

The structural basis of hair is the hair follicle, a complex miniature organ in the skin [3]. The morphogenesis of the hair follicle is initially triggered by the activation of the Wnt/β‐catenin signaling pathway in the dermis, which induces the formation of a placode in the subepidermis [4]. The placode grows downward to form the hair peg that continues to lengthen and, together with the dermal papilla, constitutes the bulb of the hair [5, 6]. At this point, the developing hair follicle starts to form different cell layers, among which the bulge area mainly stores hair follicle stem cells (HFSCs) [7]. The HFSCs are able to continuously differentiate into various follicle cells and then regrow new hairs [8]. According to previous literature, the activation of HFSCs is regulated by the Wnt/β‐catenin signaling pathway, which positively regulates the HFSCs proliferation, differentiation, and hair follicle cycle [9] (Figure 1).

The Wnt ligand binds to the Frizzled receptor before forming a complex with low‐density lipoprotein receptor‐related protein 5/6 (LRP5/6), which prevents the degradation of β‐catenin [10, 11]. The protein of β‐catenin accumulates in the cytoplasm and is subsequently translocated to the nucleus to activate the transcription of Wnt genes [12]. The Wnt/β‐catenin pathway is mainly regulated by signaling molecules in the cellular microenvironment, including miRNAs [13]. The miRNAs are very small RNA molecules, only about 20–25 nucleotides long [14]. They act like “regulatory switches” in the cell, regulating gene expression [15]. Specifically, miRNAs reduce the production of proteins by binding to specific messenger RNAs (mRNAs) and either inhibiting their translation or causing their degradation [14].

**FIGURE 2:**
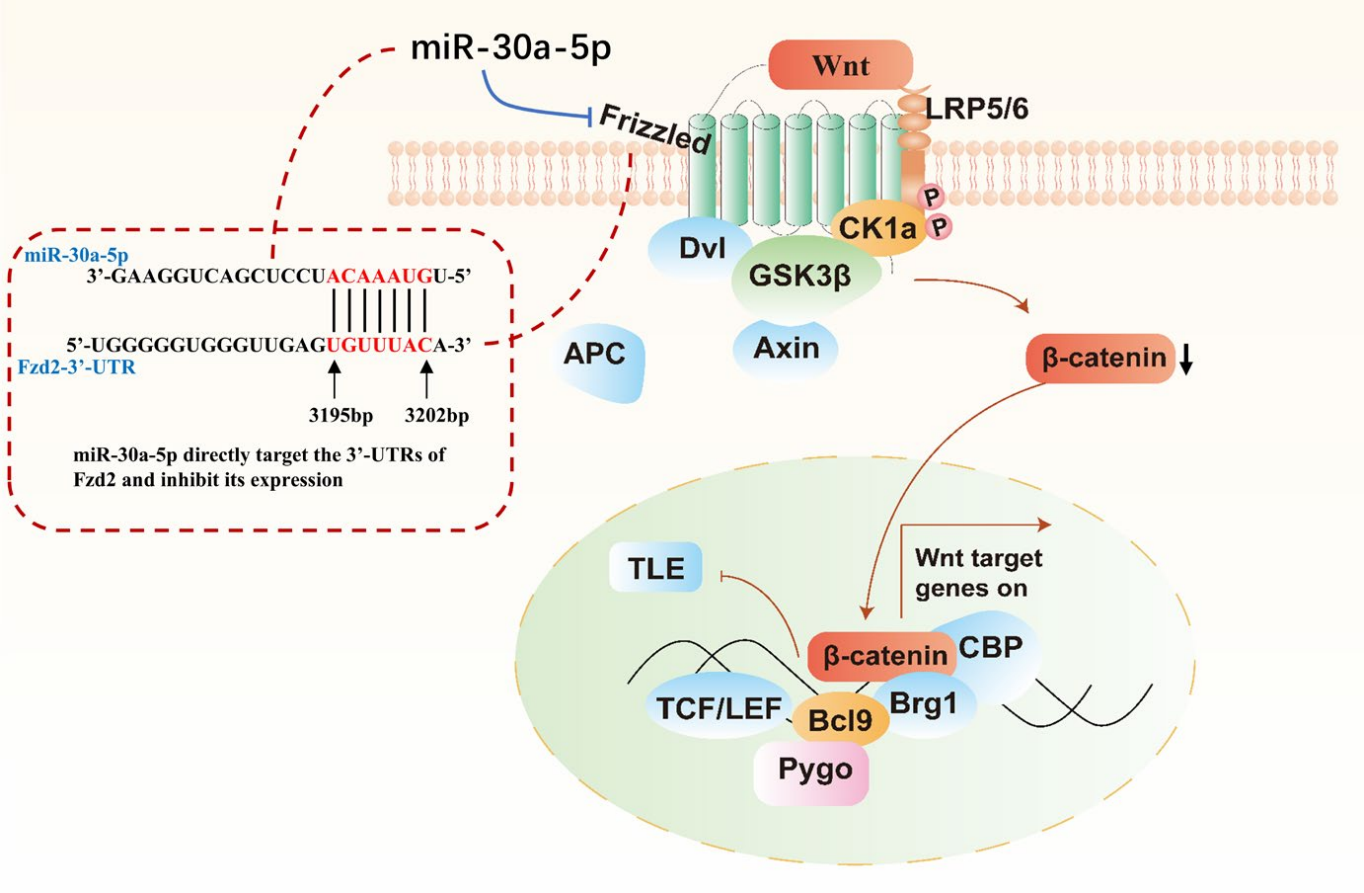
Mechanism of miR‐30a‐5p inhibiting the Wnt/β‐catenin signaling pathway. miR‐30a‐5p directly targets frizzled mRNA and negatively regulates frizzled expression by binding to the 3'‐UTR of Fzd2 mRNA, leading to degradation of β‐catenin in the cytoplasm and thus inhibiting the Wnt/β‐catenin signaling pathway.

miR‐30a is a member of the miRNA‐30 family and is widely involved in regulating gene expression [16]. In recent years, a number of miRNAs related to hair follicle growth and development have been screened using high‐throughput sequencing and bioinformatics analysis techniques [17, 18]. Ding et al. found that miR‐30a‐5p was significantly increased during the telogen by investigating the network of competing endogenous RNAs (ceRNAs) in the hair follicle cycle in mice [18]. In addition, Qi et al. demonstrated that miR‐30a‐5p can directly target the 3'‐untranslated regions (3'‐UTR) of frizzled‐2 (Fzd2), resulting in the inhibition of the Wnt/β‐catenin pathway in squamous cell carcinoma of the esophagus [19] (Figure 2).

Based on the above findings, we hypothesized that miR‐30a‐5p has perhaps an inhibitory effect on the Wnt/β‐catenin signaling pathway in HFSCs. The aim of this study was to investigate whether miR‐30a‐5p could impact on the proliferation and apoptosis of HFSCs through the Wnt/β‐catenin signaling pathway in hair follicles, providing valuable experimental data for potential clinical intervention.

## 2 Methods

### 2.1 Overall Design of the Study

**FIGURE 3:**
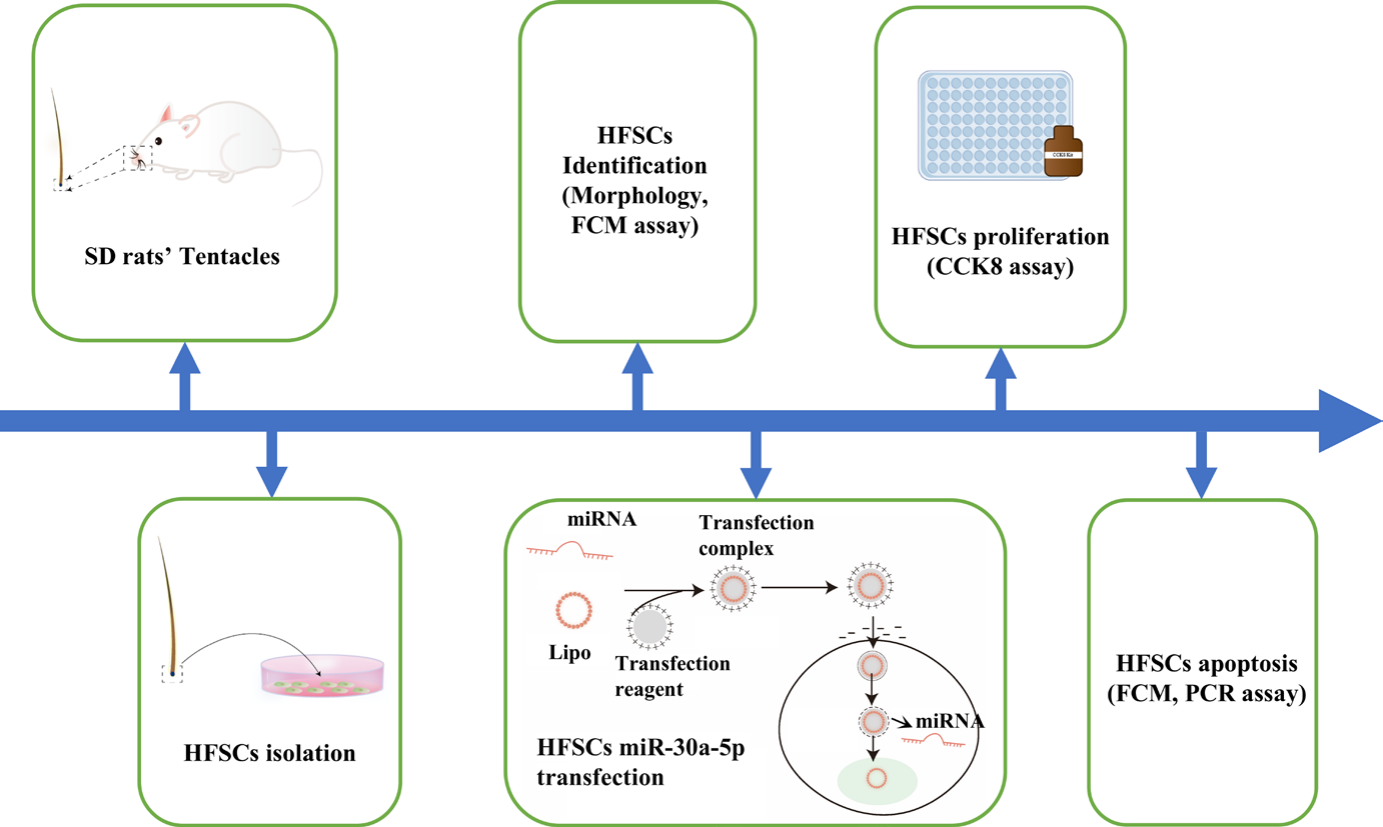
Flowchart of the general design of the study. Primary HFSCs from SD rat tentacles were validated by morphology and cell surface biomarkers. Modulation of miR‐30a‐5p expression in HFSCs by Lipofectamine approach was performed to assess alterations in cell proliferation and apoptosis. Each analysis was repeated five times of independent experiments.

HFSCs were isolated from the hair follicles of the tentacles of SD rats and identified by morphology and flow cytometry. miR‐30a‐5p mimic, miR‐NC, in‐miR‐30a‐5p, and in‐miR‐NC were subsequently transfected by the Lipofectamine method into the HFSCs. The transfected HFSCs were subjected to proliferation and apoptosis assays, and the Wnt/β‐catenin signaling pathway was analyzed (Figure 3).

### 2.2 Isolation, Culture, and Characterization of HFSCs

#### 2.2.1 Isolation and Culture of HFSCs

One‐week‐old SD rats were sacrificed with CO_2_ and sterilized by immersion in 75% ethanol. The hair follicle tissues of the tentacles were separated with ophthalmic scissors in a sterile bench, and then, the hair follicles were completely separated from the skin tissues with a syringe needle under a microscope. Subsequently, the ends of the hair follicles were cut off and only the follicular bulge was retained. The bulge was digested with a mixture of 1% collagenase IV (Merck KGaA, Germany) and 1% dispase II (Merck KGaA, Germany) at 37°C for 90 min before terminating the digestion with 10% FBS. The digested solution was filtered to remove tissue debris. After centrifugation, the resuspended digested cells were seeded into petri dishes followed by the addition of complete medium for HFSCs and cultured at 37°C under 5% CO_2_, and then the medium was changed every 2–3 days. The density and morphology of the cells were observed under the microscope every day. Animal experiments were performed according to the guidelines approved by the Laboratory Animal Committee of the University of South China (Approval number: 2021LL0601001).

#### 2.2.2 Identification of HFSCs

##### 2.2.2.1 HE Staining

HFSCs from the third passage were fixed with a mixture of methanol and glacial acetic acid, and then rinsed with deionized water for three times. Then dropwise freshly prepared hematoxylin staining solution was immersed for 2–3 min, and the nuclei of the cells were blue after being treated with differentiation solution and rinsed with tap water. Finally, the cytoplasm of the cells was stained with eosin for 1 min, before being dehydrated sequentially by 75%, 95%, and pure ethanol. The stained cells were observed under the microscope for the gross morphology of the cells.

##### 2.2.2.2 HFSC Surface Markers

Third‐passage HFSCs in logarithmic growth phase were collected, washed twice with PBS, added pre‐cooled 75% ethanol and fixed at 4°C for 30 min. After centrifugation at 1500 rpm for 5 min, the supernatant was discarded and washed twice with PBS. After resuspending 1 × 10^6^ cells with 500 μL of PBS containing 1% FBS, 1 μL of FITC anti‐integrin β1 (CD29) antibody (1: 500) (Biolegend, USA), 1 μL of FITC anti‐keratin 15 (CK15) antibody (1: 500) (Novus Biologicals, Canada) and 1 μL of PE anti‐CD34 antibody (1: 500) (Abcam, UK) were added. The liquid was gently mixed and then incubated at 4°C for 30 min under light protection. The supernatant was removed by centrifugation, and the cells were resuspended again with 500 μL of PBS, and the expression rates of the corresponding surface markers in the cell populations were detected by flow cytometry. Each cell surface marker was analyzed using 5000 events to analyze the data.

### 2.3 HFSC Transfection and Identification of Transfection Efficiency

Briefly, transfection was performed by referring to the Lipofectamine 2000 (Thermo Fisher Scientific, USA) instructions. when HFSCs reached 70% confluence, the Lipofectamine reagent was mixed with the desired miRNAs (miR‐30a‐5p mimic, in‐miR‐30a‐5p, miR‐NC, and in‐miR‐NC) (Sangon Biotech (Shanghai) Co., Ltd.) and stored at room temperature for 5 min to form the miRNA‐lipid complex. Thereafter, the complex was added to the cells and cultured in a 5% CO_2_ incubator for 24 h at 37°C.

Total RNA of transfected HFSCs was extracted with Trizol kit (Invitrogen, USA), and cDNA was amplified with miRNA 1st Strand cDNA Synthesis Kit (by stem‐loop) kit (Vazyme, China). Next, RT‐qPCR was performed using miRNA Universal SYBR qPCR Master Mix kit (Vazyme, China). Cycling conditions were: pre‐denaturation at 95°C for 5 min followed by 40 cycles of reaction (95°C, 10 s), followed by annealing treatment (60°C, 30 s). The relative expression of each target gene mRNA was calculated by the 2 (^−∆∆Ct^) method. The specific primers for the mRNAs and miRNAs of the tested genes were purchased and synthesized from Sangon Biotech (Shanghai) Co., Ltd. (Table 1).

**TABLE 1:** Primer sequences.

Gene type	Primers (5′–3′)
GAPDH	F: AACTTTGGCATTGTGGAAGG
	R: ACACATTGGGGGTAGGAACA
U6	F: GTGCTCGCTTCGGCAGCA
	R: CAAAATATGGAACGCTTC
miR‐30a‐5p	F: AACGAGACGACGACAGAC
	R: TGTAAACATCCTCGACTGGAAG
β‐catenin	F: TGAACCCCAAAGCCAACC
	R: AGAGGCGTACAGGGACAGCA
Bax	F: AGGCCTCCTCTCCTACTTCG
	R: CCTTTCCCCTTCCCCCATTC
Bcl‐2	F: GAACTGGGGGAGGATTGTGG
	R: GCATGCTGGGGCCATATAGT
PCNA	F: AAGCCACTCCACTGTCTCCT
	R:CATCCTCGATCTTGGGAGCC

### 2.4 CCK8 Detection of HFSC Proliferation

Each group of transfected HFSCs was inoculated at a density of 2 × 103 cells/cm_2_ and incubated for 24 h at 37°C in an incubator. 5 μL of CCK8 (Abcam, UK) solution was added to each well of a 96‐well plate before incubation for 4 h at 37°C. The absorbance (Ab) value at 450 nm was measured by an enzyme meter. Cell viability was calculated according to the following formula: Cell viability (%) = (Ab_2_—Ab_1_)/Ab_1_ × 100%.

### 2.5 Detection of Apoptosis by Flow Cytometry

The steps for collection of transfected HFSCs in each group were the same as §2.2.2.2. Next, 5 μL of Annexin V‐FITC (1: 100) (Thermo Fisher Scientific, USA) was added to the cell suspension and incubated for 15 min at 4°C under light protection. After PBS washing, 10 μL of PI (1: 50) (Thermo Fisher Scientific, USA) was mixed with the cell suspension and kept at 4°C for 5 min. For PBS resuspended cells immediately before detection by flow cytometry, excess dye was washed with PBS.

### 2.6 Statistical Analysis

All data were statistically analyzed using GraphPad Prism (GraphPad Software, USA) and data were expressed using mean ± standard deviation. Comparisons between multiple groups were analyzed using the one‐way ANOVA test, with a *p* value < 0.05 to consider a statistically significant difference.

## 3 Results

### 3.1 Identification of HFSCs

**FIGURE 4:**
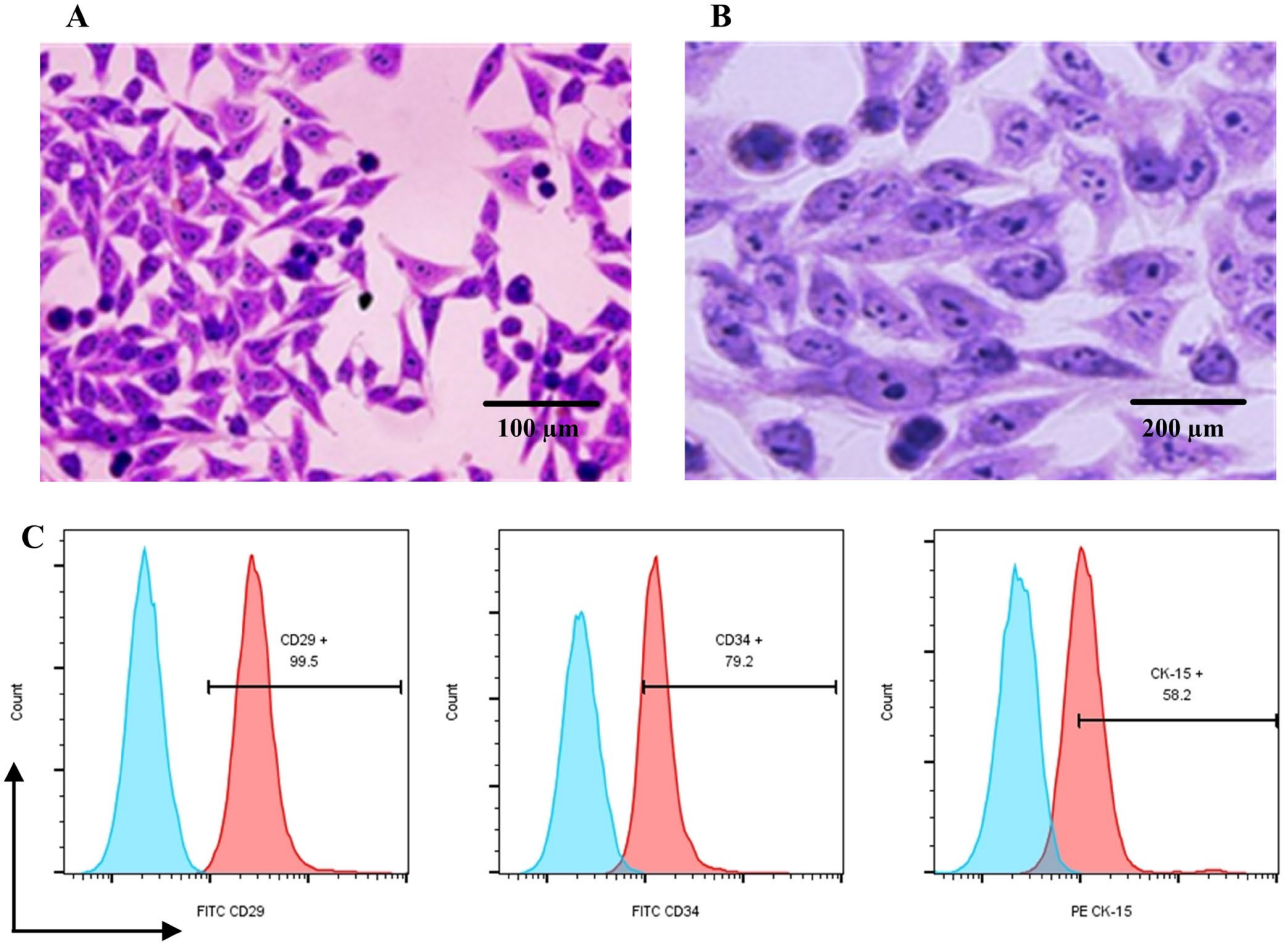
Morphological observation of HFSCs and identification of surface markers. (A) HE staining of third passage HFSCs showed a typical “curly eye” cell morphology. (B) Observation of the cell morphology under high magnification microscope. (C) Detection of HFSCs' surface markers by flow cytometry revealed that the expression rates of CD29, CD34, and CK‐15 were 99.5%, 79.2%, and 59.2%, respectively. *n* = 5.

Gross morphological observation revealed that the third generation of HFSCs showed a typical morphology with a typical “curly eye” shape (Figure 4A,B). At the same time, the analysis of the surface markers of HFSCs showed that CD34, integrin β1 (CD29) and CK15 were highly expressed (Figure 4C).

### 3.2 Changes in Viability of Transfected HFSCs in Each Group

**FIGURE 5:**
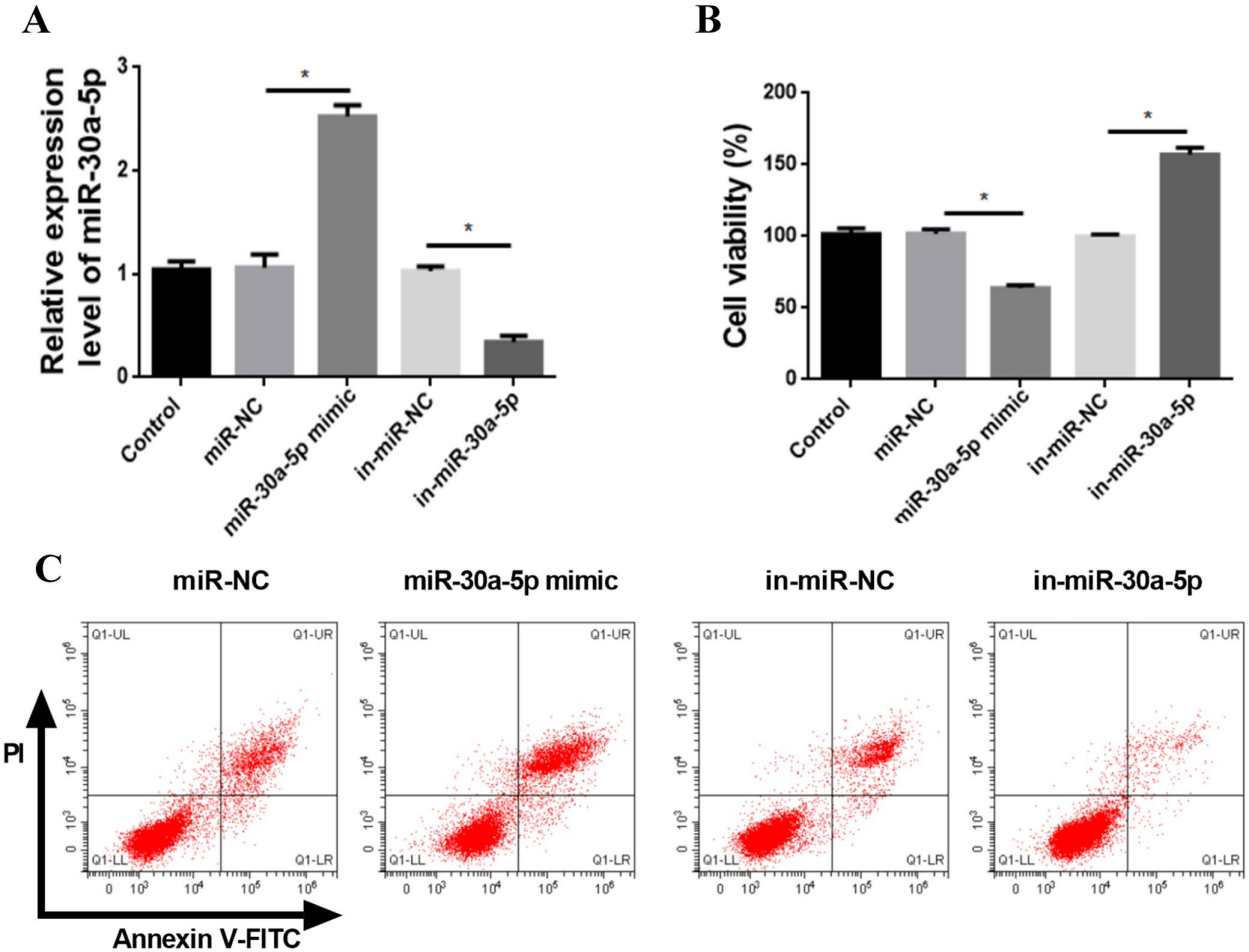
Effect of miR‐30a‐5p on HFSC viability. (A) Expression alteration of miR‐30a‐5p in HFSCs of each group after transfection. (B) Proliferation changes of HFSCs in each group after miR‐30a‐5p transfection. (C) Apoptosis levels of HFSCs after miR‐30a‐5p transfection. *p < 0.05, *n* = 5.

The results of cell transfection efficiency confirmed that the expression of miR‐30a‐5p mimic group was significantly higher compared with miR‐NC group (*p* < 0.05); while the expression of in‐miR‐30a‐5p group was notably lower compared with in‐miR‐NC group (*p* < 0.05) (Figure 5A). And, both miR‐NC and in‐miR‐NC groups were not statistically different from control group (*p* > 0.05) (Figure 5A).

Cell proliferation analysis revealed that HFSC proliferation ability was obviously reduced in the miR‐30a‐5p mimic group compared with miR‐NC (*p* < 0.05). On the contrary, the cell proliferation rate in the in‐miR‐30a‐5p group was clearly upregulated compared with in‐miR‐NC (*p* < 0.05) (Figure 5B).

The apoptosis rate showed a pronounced increase in the miR‐30a‐5p mimic group and a marked decrease in the in‐miR‐30a‐5p group (Figure 5C).

### 3.3 Transfected HFSCs in Each Group Regulated the Expression of Genes Associated With Cell Viability

**FIGURE 6:**
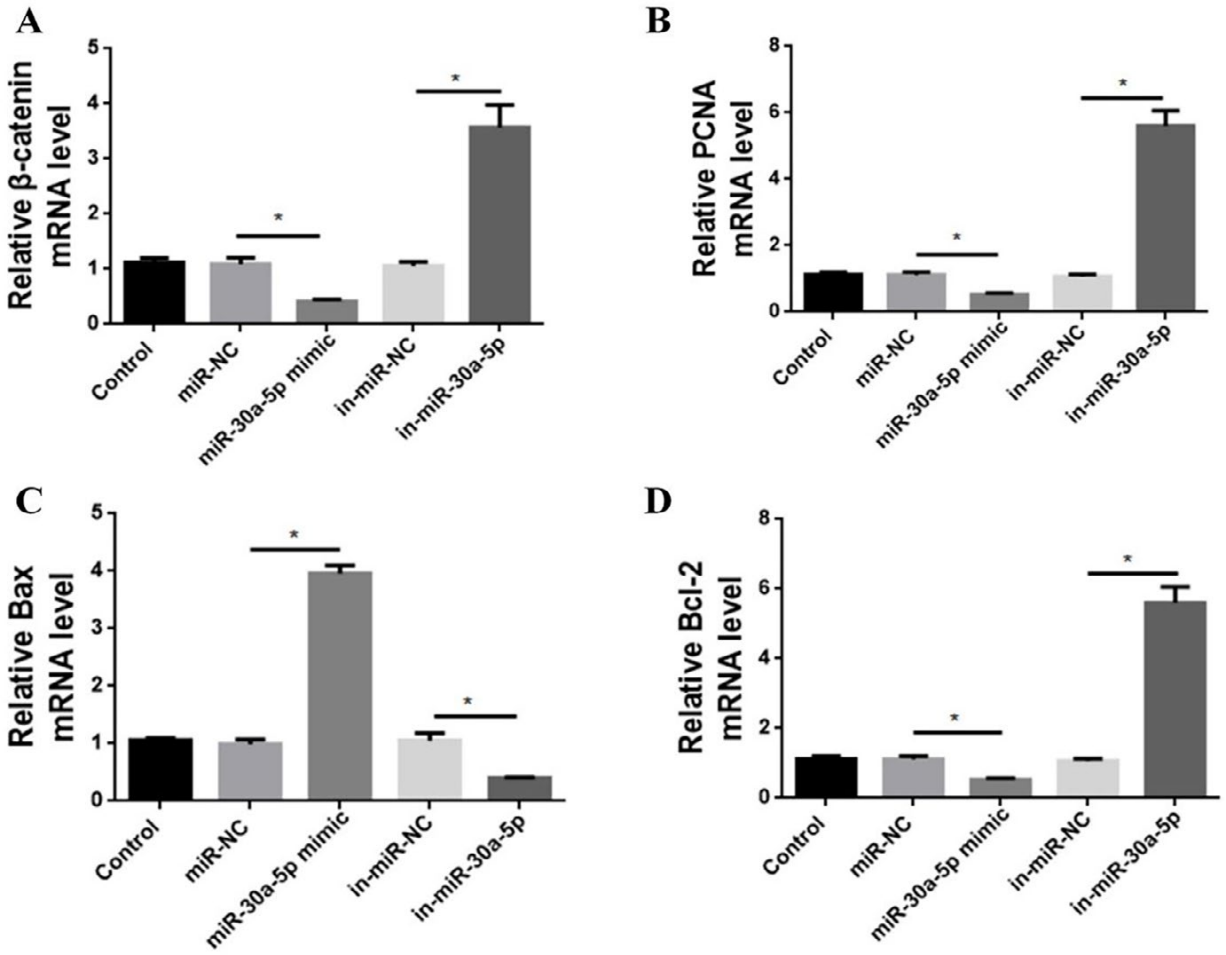
MiR‐30a‐5p effects on the expression of corresponding genes in HFSCs. (A) Expression of β‐catenin mRNA in HFSCs. (B) Expression of PCNA mRNA in HFSCs. (C) Expression of Bax mRNA in HFSCs. (D) Expression of Bcl‐2 mRNA in HFSCs. *p < 0.05, *n* = 5.

Analysis of mRNA transcript levels of β‐catenin revealed that the miR‐30a‐5p mimic group dramatically repressed β‐catenin mRNA transcription compared to the miR‐NC group (*p* < 0.05). The expression of in‐miR‐30a‐5p group was significantly elevated compared with in‐miR‐NC group (*p* < 0.05) (Figure 6A).

Similarly, the results of mRNA expression of PCNA showed a marked decrease in the miR‐30a‐5p mimic group compared to the miR‐NC group (*p* < 0.05). In the in‐miR‐30a‐5p group, the mRNA transcript levels of PCNA were obviously upregulated compared with the in‐miR‐NC group (*p* < 0.05) (Figure 6B).

Bax and Bcl‐2 mRNA expression assays confirmed that overexpression of miR‐30a‐5p resulted in a distinct increase in the mRNA expression of Bax (*p* < 0.05), whereas the mRNA transcript level of Bcl‐2 was significantly reduced (*p* < 0.05) (Figure 6C,D).

## 4 Discussion

Currently, the main treatments for hair loss are medications and surgical hair transplants [20]. Long‐term medications for hair loss can either cause side effects or their effectiveness gradually diminishes [21]. In addition, hair transplant surgery is not the best treatment option for hair loss patients because invasive treatments may destroy hair follicles in the resting phase [22]. If resting hair follicles can be induced to enter the anagen phase, hair loss patients can still grow normal hair [23].

SD rats are one of the popular experimental critters for hair follicle drug studies, hair loss modeling, and other applications [24]. Rat vibrissa follicles are much larger compared to normal hair follicles, which is easy to observe and manipulate for research, and the growth cycle of multiple hair follicles is synchronized [25]. HFSCs from rat vibrissa can differentiate into many different types of cells, including those that make up hair, such as keratinocytes, pigment cells, and follicular epithelial cells [26]. When there is injury to the hair, HFSCs can repair the damaged hair follicle tissue and regrow hair through proliferation, migration and differentiation [27]. After staining, the extracted HFSCs showed a typical flat polygonal or “paving stone” morphology [28]. Moreover, the surface markers of HFSCs detected by flow cytometry showed that CD34, integrin β1 (CD29), and CK15 were all highly expressed. These criteria were consistent with the reported characteristics of HFSCs [29].

miRNAs have regulatory functions on cellular life activities, including cell proliferation, differentiation, senescence, and apoptosis [30]. miR‐30a‐5p, as a member of the miR‐30 family, is widely present in a variety of cells, and is closely related to the processes of cell proliferation, apoptosis, migration, and invasion [31]. In this experiment, based on the detection of cell activity and proliferation or apoptosis related genes, we found that upregulation of miR‐30a‐5p can cause a decrease in cell proliferation, and at the same time, the cell proliferation gene PCNA expression also appear to be decreased accordingly. Currently, most of the studies on miR‐30a‐5p are in the direction of cancer. miR‐30a‐5p effect on lung squamous cell carcinoma proliferation, Zeng et al. found that high expression of miR‐30a‐5p caused a decrease in proliferation‐regulated protease cyclin‐dependent kinase 4 (CDK4) [32]. The results of the apoptosis assay in this study showed that the rising expression of miR‐30a‐5p led to an increase in apoptosis rate, with a corresponding increase in intracellular expression of the pro‐apoptotic Bax gene, and conversely a decrease in the level of the anti‐apoptotic Bcl‐2 gene. The same trend was found in the study of chronic heart failure in rats by Wu et al. [31]. Therefore, the above experimental results can reliably confirm that miR‐30a‐5p inhibits the proliferation of HFSCs.

The Wnt/β‐catenin signaling pathway maintains the ability of dermal papilla (DP) cells to induce hair regrowth and promotes hair follicles into the anagen phase [33]. No study has elucidated the association between miR‐30a‐5p and the Wnt/β‐catenin signaling pathway in HFSCs. During cell proliferation, previous studies have found that both miR‐30a‐5p and Wnt/β‐catenin are mostly negatively correlated, such as the miR‐30a‐5p/chromodomain helicase DNA binding protein 1 (CHD1) axis, which, through inhibition of the Wnt/β‐catenin signaling pathway inhibits the proliferation, migration and invasion of ovarian cancer cells to achieve apoptosis [34]. Therefore, we can speculate that the inhibitory effect of miR‐30a‐5p on Wnt/β‐catenin signaling pathway may exist in HFSCs. To verify this hypothesis, miR‐30a‐5p mimic group was found to significantly inhibit β‐catenin expression in HFSCs by PCR. This indirectly confirmed our speculation that miR‐30a‐5p is an inhibitory link to the Wnt/β‐catenin signaling pathway. If the Wnt/β‐catenin signaling pathway is downregulated, miR‐30a‐5p would cause a decrease in the proliferative capacity of HFSCs and an increase in the level of apoptosis, which ultimately results in a weakening or even cessation of the transition of the hair follicle from the resting phase to the anagen phase. However, Tan et al. found that high miR‐30a‐5p expression promoted the proliferation of human pulmonary artery endothelial cells (HPAEC) under hypoxic conditions of pulmonary arterial hypertension (PAH) [35]. The reason for this is that YKL‐40 (also known as chitinase 3‐like 1) causes a decrease in HPAEC proliferation, whereas the inhibitory effect of miR‐30a‐5p on YKL‐40 promotes HPAEC proliferation and inhibits apoptosis [36]. Thus, miR‐30a‐5p plays different roles in different diseases. When miR‐30a‐5p is highly expressed, it promotes the proliferation of HPAEC in PAH and inhibits the proliferation of HFSCs in the hair follicle cycle.

For future clinical applications, miR‐30a‐5p as a potential therapeutic target for hair loss requires an effective drug carrier. Hydrogel microneedle is a biocompatible microneedle array made of hydrogel material [37]. It combines the characteristics of microneedle and hydrogel, and is able to penetrate the epidermis and even the dermis of the skin to deliver drugs or bioactive substances to specific layers of the skin in a minimally invasive manner, and the piggybacked drugs can be released in a controlled manner [38]. Therefore, hydrogel microneedle, as a novel miR‐30a‐5p inhibitor delivery system, can effectively overcome the many shortcomings of the traditional hair loss treatment program, and provide a minimally invasive, precise, and long‐lasting treatment for hair loss patients. In the future, with the development of material science and microfabrication technology, hydrogel microneedle is expected to become one of the mainstream technologies in hair loss treatment.

Despite advancements in research and development, miRNA therapy still faces significant challenges in the transition from preclinical to clinical applications. To date, the clinical application of miRNAs has been primarily limited to the utilization of their biomarkers for the purpose of assisting in the diagnosis of diseases [39]. The application of miRNAs in the context of disease treatment remains in the phase of clinical trials [40]. So far, the US Food and Drug Administration and the European Medicines Agency have only approved a limited number of miRNA therapies for use in small‐scale clinical trials [41]. At present, phase II clinical trials are underway for the following programs: MRG‐110, which targets miR‐92a, is being investigated for its potential in treating wounds. MesomiR 1, which targets miR‐16, is being explored for its efficacy in treating non‐small cell lung cancer. CDR132L, which targets miR‐132, is being considered for its ability to improve heart failure outcomes. Remlarsen/MRG‐201, which targets miR‐29, is being evaluated for its potential in treating keloid disorders. Miravirsen/SPC3649, which targets miR‐122, is being investigated for its ability to improve outcomes in patients with chronic hepatitis C virus infection [14]. It is evident that miRNAs exert a profound influence on genetic regulation. Individual miRNAs can orchestrate entire cellular pathways by interacting with a vast array of target genes [42]. However, this multifaceted capacity also presents a challenge, as it makes off‐targeting a significant concern [43]. Despite recent advancements, it is clear that we are still in the early stages of developing miRNA‐based therapies.

The present study provides a new perspective on the application of miR‐30a‐5p as a therapeutic target for hair loss, but there are still some limitations. First, the effects of miR‐30a‐5p at the cellular level in vitro in this study cannot be directly generalized to the more complex in vivo environment. The hair follicle, as a complex microorgan, has physiological activities that are co‐regulated by multiple signaling pathways and cell types [27]. Therefore, we need to develop animal models based on gene editing technology to further study the functional role of miR‐30a‐5p at a holistic level, so as to fully elucidate its molecular mechanism in hair follicle regeneration and regulation. Second, the present study indirectly demonstrated that miR‐30a‐5p may affect the function of HFSCs by regulating the Wnt/β‐catenin signaling pathway through the alteration of β‐catenin expression. However, this evidence is still weak. We hypothesized that miR‐30a‐5p may act as a negative regulator of the frizzled receptor and thus inhibit the activation of the Wnt signaling pathway, but this hypothesis is mainly based on indirect evidence in the previous literature and lacks direct validation in HFSCs. Therefore, subsequent studies should further investigate whether miR‐30a‐5p functions by regulating Frizzled or other Wnt signaling pathway‐related factors. Meanwhile, we should also pay attention to whether there are other potential target genes of miR‐30a‐5p and reveal its mechanism of action through systematic target validation experiments (e.g., RNA immunoprecipitation, dual luciferase reporter system, etc.) [44]. In addition, miR‐30a‐5p is only one member of the miR‐30 family, and other miRNAs in the miR‐30 family (e.g., miR‐30b, miR‐30c, etc.) may also play important roles in the regulation of hair follicle growth and may affect the proliferation of HFSCs through other signaling pathways (e.g., VEGF, PI3K‐Akt, or MAPK) [45]. Complex interactions and redundancy mechanisms exist among different miRNAs, and therefore, only studying the single role of miR‐30a‐5p may not be able to fully reveal the regulatory network of this family in hair loss. Therefore, future studies should systematically investigate the interrelationships among miR‐30 family members and their synergistic effects in hair follicle regeneration.

Overall, the above findings not only help to further understand the mechanism of miR‐30a‐5p in the cyclic development of hair follicles, but also have the potential to be a biomarker for determining hair loss or a new target for intervention, providing reliable experimental evidence for the development of new diagnostic and therapeutic approaches.

## 5 Conclusion

Downregulation of miR‐30a‐5p activates the Wnt/β‐catenin signaling pathway to inhibit apoptosis and promote proliferation of HFSCs.

## Author Contributions

Risheng Wang, Wentao Wu, and Jinwei Chen performed the research. Risheng Wang and Nian Chen designed the research study. Xiangping Xu, Chiyu Jia, and Nian Chen contributed essential reagents or tools. Wentao Wu, Jinwei Chen, and Risheng Wang analyzed the data. Yiping Wang wrote the paper.

## Ethics Statement

This study protocol was approved by the Medical Ethics Committee of the University of South China (Processing number: 2021LL0601001).

## Conflicts of Interest

The authors declare no conflicts of interest.

## Supporting information


Data S1.

## Data Availability

The data that support the findings of this study are available from the corresponding author upon reasonable request.
