# Supplementary material for: Assessment of the Role of miR‐30a‐5p on the Proliferation and Apoptosis of Hair Follicle Stem Cells
Source: J Cosmet Dermatol. 2024 Oct 23;24(1):e16644. doi: 10.1111/jocd.16644 (PMC11743330; doi:10.1111/jocd.16644)
Supplement: Supplementary file 1 — Data S1. [file JOCD-24-e16644-s001.docx]

Collagenase IV (Merck KGaA, C4-BIOC)

Dispase II (Merck KGaA, 04942078001)

Hematoxylin (Merck KGaA, 517-28-2)

Eosin (Merck KGaA, HT110116)

Ethanol (Merck KGaA, 1.00983)

PBS (Vazyme, 101-01)

Complete medium for HFSCs (immocell, IMP-R185-1)

FBS (Merck KGaA, 12103C)

FITC anti-integrin β1 (CD29) antibody (Biolegend, 102205)

FITC anti-Cytokeratin 15 (CK15) antibody (Novus Biologicals, NBP2-54463F)

PE anti-CD34 antibody (Abcam, ab223930)

Lipofectamine® 2000 (Thermo Fisher Scientific, 11668019)

Trizol kit (Invitrogen, 12183555)

miRNA 1st Strand cDNA Synthesis Kit (by stem-loop) kit (Vazyme, MR101)

miRNA Universal SYBR qPCR Master Mix kit (Vazyme, MQ101-02)

CCK8 (Abcam, ab228554)

Annexin V-FITC (Thermo Fisher Scientific, V13242)

PI (Thermo Fisher Scientific, V13242)

**miR-30a-5p inhibitor design and synthesis:**

The miR-30a-5p inhibitor used in this study is a chemically synthesized antisense oligonucleotide (ASO) specifically designed to bind to the mature miR-30a-5p sequence and prevent its interaction with target mRNAs.

The sequence was designed based on the mature miR-30a-5p sequence (5’UGUAAACAUCCUCGACUGGAAG3’) obtained from the miRBase database. The miR-30a-5p inhibitor (AntagomiR: 5’C*U*UCCAGUCGAGGAUGUU*U*A*C*A3’, all bases were 2-OMe base, *: phosphorothioate) was synthesized by **Sangon Biotech (Shanghai, China)**.
